# Bronchioalveolar carcinoma in an adult alpaca (*Vicugna pacos*)

**DOI:** 10.1186/s12917-019-1895-8

**Published:** 2019-05-09

**Authors:** Lara Moser, Kristel Kegler, Christina Precht, Patrik Zanolari

**Affiliations:** 1 Clinic for Ruminants, Department of Clinical Veterinary Medicine, Vetsuisse-Faculty, University of Bern, Bremgartenstrasse 109a, 3012 Bern, Switzerland; 2 Institute for Veterinary Pathology, Vetsuisse-Faculty, University of Bern, Länggassstrasse 122, 3012 Bern, Switzerland; 3 Clinical Radiology, Department of Clinical Veterinary Medicine, Vetsuisse-Faculty, University of Bern, Länggassstrase 124, 3012 Bern, Switzerland

**Keywords:** South American camelid, Neoplasia, Bronchiolar adenocarcinoma, Lung CT-scan, Radiology, Alpaca

## Abstract

**Background:**

This report describes a case of a bronchiolar adenocarcinoma in a 6-year old alpaca mare. For the first time in an alpaca, neoplasia was classified by histopathology as a lepidic-predominant bronchiolar adenocarcinoma.

**Case presentation:**

The mare was referred to the Clinic for Ruminants after a 6-week period of forced breathing and weight loss. The clinical examination included complete blood count, blood chemistry, ultrasound, radiographs and a CT-scan of the thorax. A bilateral pneumothorax and several, structures within the lung parenchyma were diagnosed. Differential diagnosis included neoplasia, tuberculosis and fungal granulomas. The owner requested euthanasia due to the mare’s ongoing deterioration. At postmortem examination, the granulomatous changes in the lungs were histopathologically classified as lepidic dominant bronchiolar adenocarcinoma.

**Conclusions:**

Neoplastic diseases are more often seen in South American camelids compared to other farm animal species. The use of a CT scan was helpful in classifying the lung lesions and give a clear prognosis.

## Background

This report describes the case of a 6-year-old non-pregnant alpaca *(Vicugna pacos)* mare of the huacaya type that was referred to the Clinic for Ruminants. According to literature [1], neoplasia is relatively common in South American camelids (SAC). A relatively high percentage of SAC are kept as pets [2], they have therefore possibly a relatively long lifespan in comparison to other ruminating species. This longer life span may contribute to a higher prevalence of neoplastic disease [1]. Although there are some reports of neoplasia associated with the respiratory system in SAC [3–5], none of them have described a diagnosis in a living animal. Because of the animal’s common pet status, the owners of SAC are often more willing to accept higher costs for diagnostic investigation and therapy when issues arise, compared to owners of other farm animal owners. To satisfyingly respond to these interests, it is important to establish expert knowledge in advanced diagnostic options as presented in this case - especially to make an exact diagnosis and provide a prognosis.

## Case presentation

### History

A 6-year old domestic huacaya alpaca non-pregnant mare with a body mass of 53 kg, was referred to the Clinic for Ruminants, Vetsuisse-Faculty, University of Bern, Switzerland for further diagnostic investigation. For 6 weeks, the mare had shown respiratory symptoms such as forced breathing but with no fever. Although the mare always had a good appetite, significant weight loss occurred during this time. A treatment by the referring veterinarian did not improve the symptoms.

### Clinical findings

At clinical examination, the alpaca was alert, nervous and in poor general condition. Body condition was moderate (bodyweight 53 kg: reference range55-90 kg) with a body condition score of 1 out of 5 (body condition score of the Australian Alpaca Association [6]). Its rectal temperature was 38.3 °C (reference range: 37.5–38.9 °C), heart rate 72 beats per minute (reference range: 60-80beats per minute) and respiratory rate 60 breaths per minute (reference range: 10–30 breaths per minute). The mare showed dyspnea with an abdominally reinforced breathing and bilateral dilated nostrils. There was no spontaneous nor provoked cough or evidence of nasal discharge. Auscultation of its lungs revealed bilateral ventrally scratching and crackling sounds, on the left dorsal aspect respiration sounds were focally absent. Further clinical examination showed no abnormalities, the alpaca had a good appetite once back in the stable, although dyspnea was still present.

Clinicopathological tests included a complete blood cell count and a blood chemistry panel. A neutrophilia (9.37 × 10^9^/l, reference interval: 3.4–9.1 × 10^9^/l) with left shift was present. Blood chemistry showed low potassium (3.64 mmol/l, reference interval: 4–5.2 mmol/l) and magnesium (0.71 mmol/l, reference interval: 0.8–1.1 mmol/l) values. Parasitological examination of a fecal sample revealed no endoparasite eggs or lungworm larvae.

### Radiographic examination

Ultrasonographic examination (Esaote piemedical®, 10mHz linear probe) of the lungs revealed on the day of admission several comet-tail-artefacts over the whole lung field. The left dorsal lung surface was focally retracted and a moderate amount of gas was visualized in the pleural space. An area of increased density of the lung parenchyma was visible. There was no evidence of pleural effusion.

**Fig. 1:**
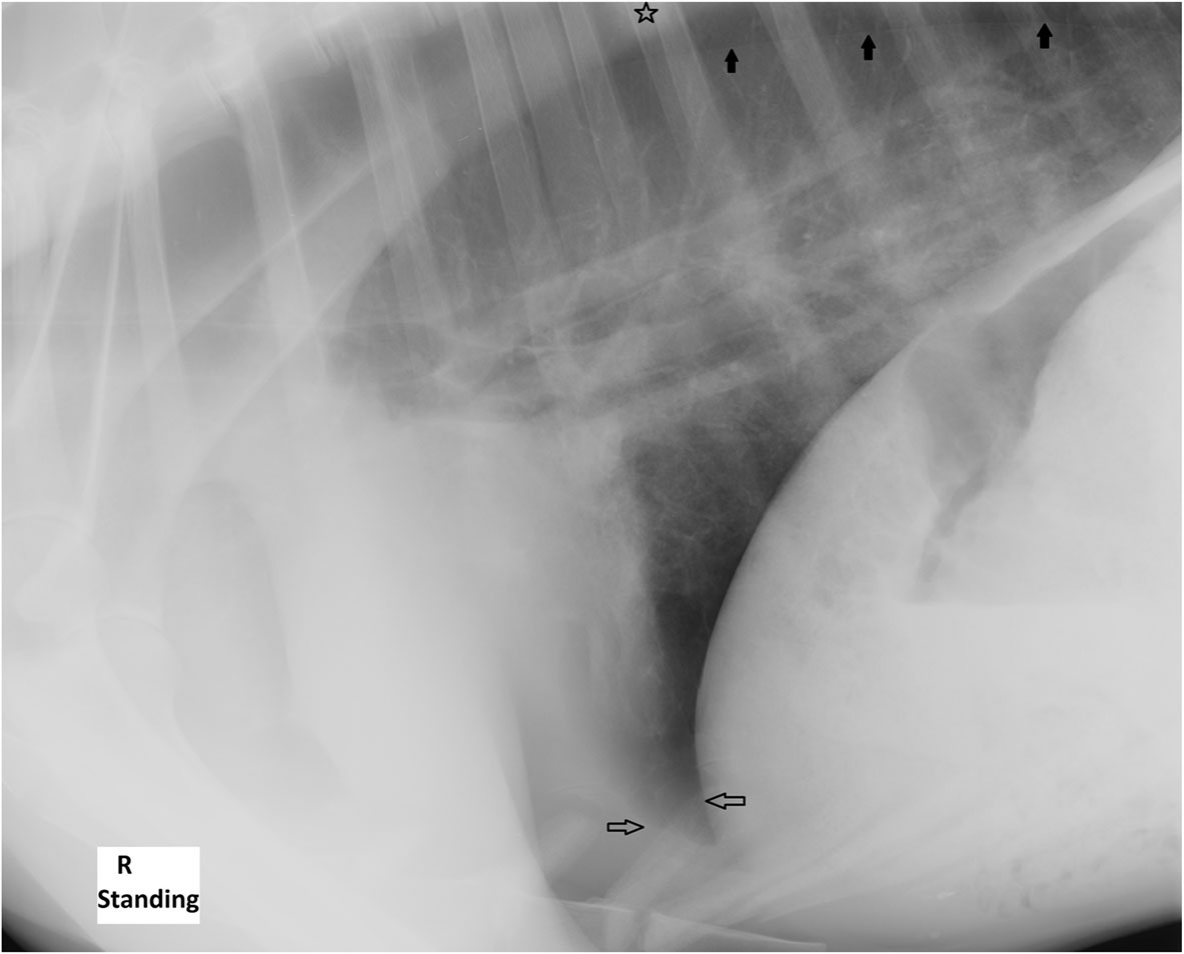
Day 1 laterolateral alpaca lung radiograph. Retracted lung lobes due to pneumothorax (black arrows) and a poorly delineated complex focal lesion in the less retracted caudal lung lobe with a focal indentation of the surface (star). Focal effacement of the contours of the diaphragm and the cardiac silhouette (white arrows)

**Fig. 2:**
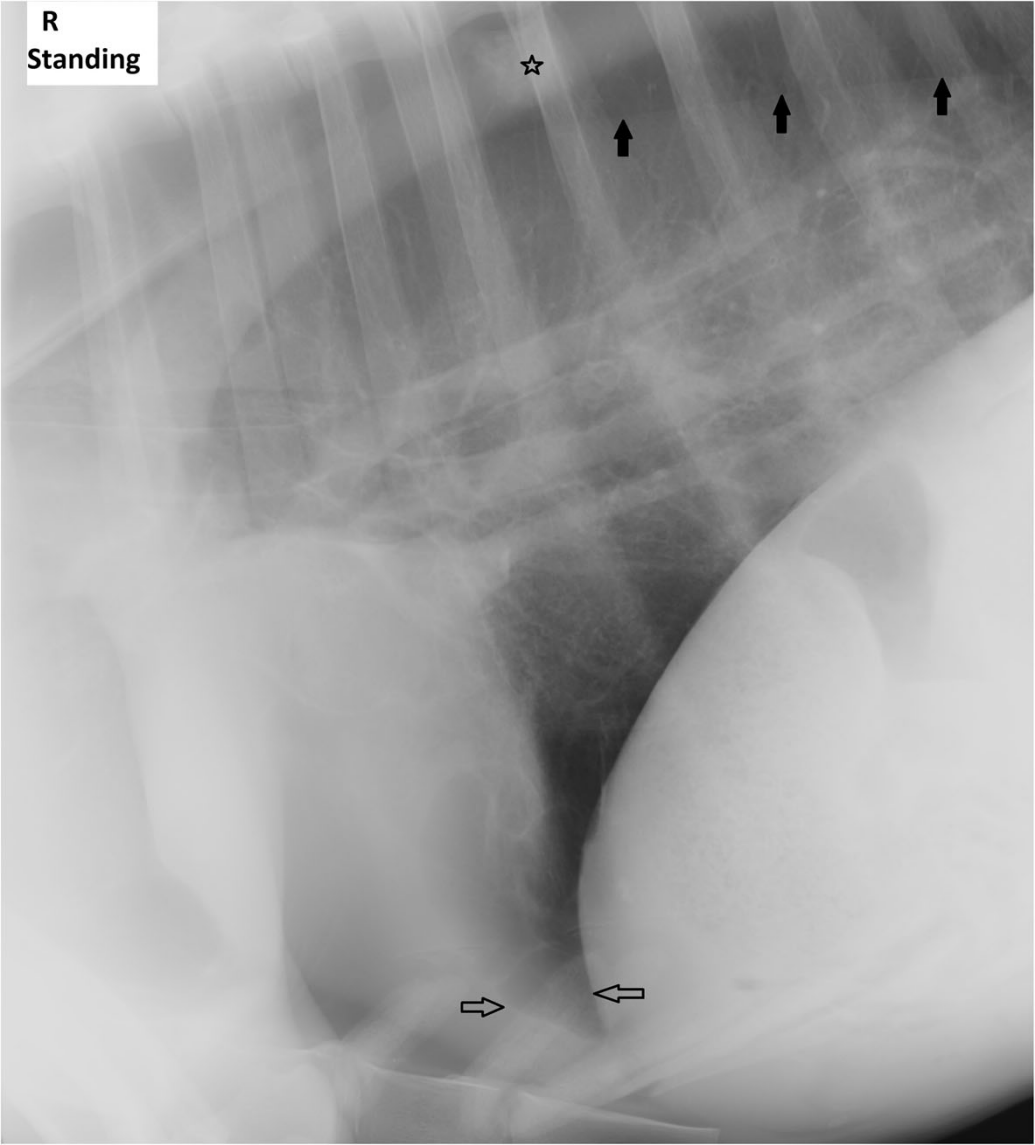
Day 4 laterolateral alpaca lung radiograph. Retracted lung lobes due to pneumothorax (black arrows) are almost unchanged to day 1. The poorly delineated complex focal lesion in the less retracted caudal lung lobe (star) is more visible due to a slightly wider frame of the radiograph. Stripy increased opacities in the cardiophrenic angle (white arrows)

Laterolateral radiographs of the lungs were taken on the day of admission (Fig. 1) and four days after admission (Fig. 2). On both occasions, a bilateral pneumothorax with retracted lung lobes, mild on one and moderate on the other side, was diagnosed. Due to a mild retraction of lung lobes in the cardiophrenic angle in combination with focal effacement of the contours of the diaphragm and the cardiac silhouette, a very mild amount of pleural effusion was suspected. A focal lesion (17x22mm) located dorsally in the less retracted caudal lung lobe was observed with ill-defined borders, associated with a focal indentation of the lung surface. In the dorsal part of the lung field, several small gas lucent (6-15 mm diameter) lesions without a wall were suspected. In the central parts of the lung field, the opacity was diffusely increased with an ill-defined mixed reticular and bronchial pattern. However, it remained unclear, if this represented a pathology or was a consequence of partially collapsed lungs due to the pneumothorax. In the follow-up radiographs four days after admission, stripy increased opacities were observed in the cardiophrenic angle (Fig. 2).

Differential diagnosis included neoplasia, fungal granulomas or tuberculosis. To obtain a better characterization of the lung pathology, a computed tomography scan(CT-scan) of the lungs in general anesthesia was performed.

**Fig. 3:**
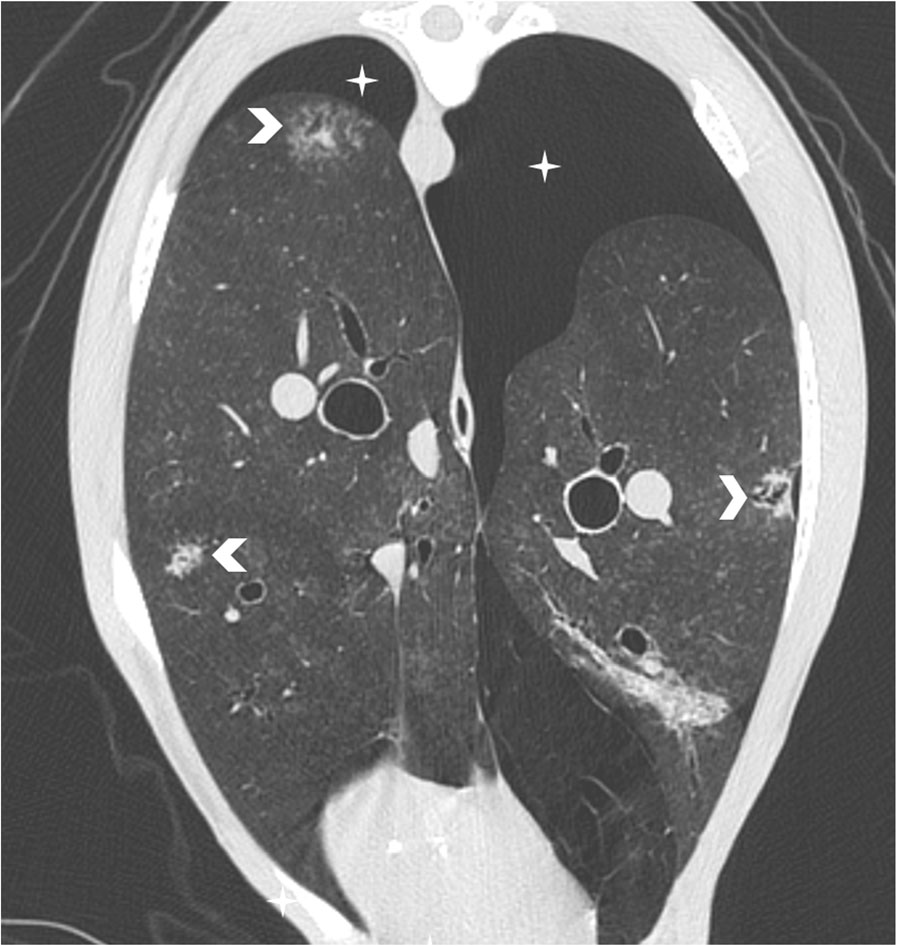
Transverse CT image of the lung showing multiple focal bronchocentric part-solid spiculate nodules with internal air bronchograms (arrowheads) in all lung lobes. The pneumothorax (star) was confirmed

**Fig. 4:**
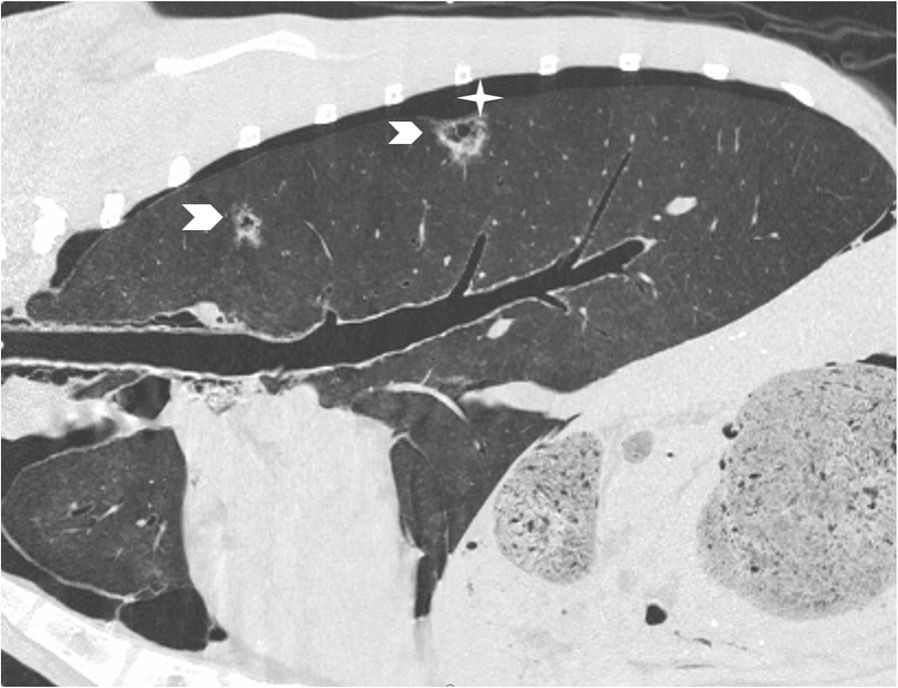
Parasagittal reconstructed CT image of the lung. Again showing multiple focal bronchocentric part-solid spiculate nodules with internal air bronchograms (arrowheads). Pneumothorax (star)

The CT-scan revealed multiple focal bronchocentric part-solid spiculate nodules with internal air bronchograms in all lung lobes. The pneumothorax was confirmed. [Figures. 3 and 4].

### Clinical evolution

Based on history, physical examination, available laboratory results and diagnostic imaging the main differential diagnosis was a pulmonary neoplasia. To distinguish between neoplasia and the other differentials as fungal granulomas or infectious agents (i.e. tuberculosis), the option of performing a fine needle aspiration or a biopsy was discussed.

Over 4 days of hospitalization, the general state and respiratory distress of the animal worsened progressively. Therapeutic options like undertaking pleural drainage to restore the pneumothorax were also discussed, but due to the poor prognosis, the owners elected for euthanasia of the alpaca. The animal was euthanized with an intravenous injection of pentobarbital (Esconarkon ad us.vet., Streuli Pharma AG, Uznach, Switzerland) in a dosage of 150 mg/kg bodyweight.

### Necropsy findings

The postmortem examination, revealed the following findings: Affecting the cranial and caudal lung lobes there were multiple, approximately 1 cm × 0.5 cm × 0.5 cm, nodular, poorly demarcated, non-encapsulated, whitish to yellowish masses. No extra-pulmonary involvement was observed. There were multifocal areas of bullous emphysema within dorsally parts of the right and left cranial lobes. Other remarkable gross findings were multifocal, 1 cm in diameter nodular, centrally mineralized parenchymal masses in the liver and multifocal mucosal ulceration within compartment 3 of the forestomaches. The other examined organs were macroscopically unremarkable and no pathological alteration could be detected.

### Histopathological findings

Samples of the lung and liver were fixed in 10% neutral buffered formalin for histological examination. The samples were routinely processed; paraffin wax embedded, stained with hematoxylin and eosin (HE), sections of 3 μm were cut and observed by standard light microscopy for histological examination.

**Fig. 5:**
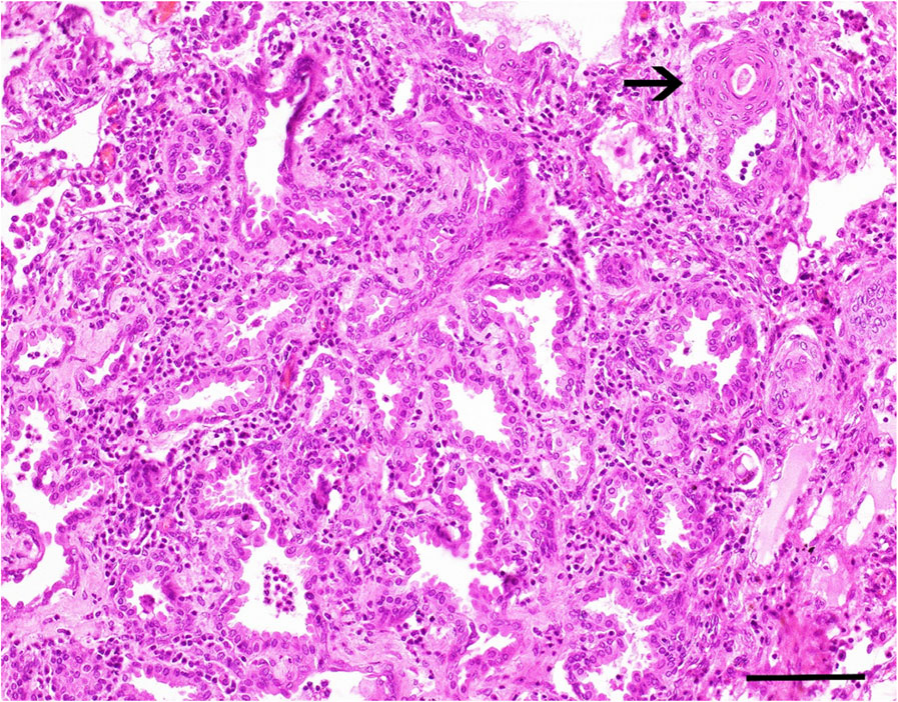
Photomicrograph of the lung of an adult alpaca with bronchioalveolar adenocarcinoma. H & E stained section of the lung shows a predominantly lepidic growth pattern along pre-existing alveolar walls and abundant fibrous stroma. Note an area of squamous epithelial cells differentiation (arrow). Bar, 100 μm

Microscopically, the pulmonary nodules consisted of unencapsulated, poorly demarcated and infiltrative growing neoplastic masses. The epithelial neoplastic masses showed lepidic growth along pre-existing alveolar walls, which was cuboidal to low columnar with mostly distinguished cell borders, scant to moderate amounts of eosinophilic cytoplasm, mostly basally placed, round nuclei with coarse-stippled chromatin pattern and up to 1 nucleoli. The stroma separating the neoplastic acinar structures was abundant and poorly cellular consisting of plump spindle cells and extracellular deposition of collagen. Occasionally, there were areas of squamous epithelial cells differentiation with central keratin pearls and no apparent acinar formation [Fig. 5]. Mitoses were very rare. Multifocal small areas of necrosis were present.The adjacent lung parenchyma showed a diffuse thickening of the alveolar septum characterized by infiltration with small numbers of lymphocytes, plasma cells and scattered macrophages. Alveolar spaces were emphysematous or multifocal filled with small numbers of foamy macrophages and few neutrophils.

The liver lesions were histologically compatible with a chronic, granulomatous and eosinophilic cholangiohepatitis. No trematodes could be observed within the examined sections and Ziehl-Neelsen-stain was negative. There was no evidence of fungal organism in the histopathological examination of liver and lung samples.

## Discussion and conclusions

Reported tumors in llamas and alpacas include fibroma/fibropapilloma, carcinoma, adenocarcinoma, lymphoma, fibrosarcoma, lipoma, melanocytoma, leiomyosarcoma and other neoplasia [3–5, 7]. The described case is the first report of a diagnosed, lepidic-predominant bronchioalveolar carcinoma in an alpaca. One case report of a bronchioalveolar carcinoma in a llama was reported by Ramos-Vara [3]. In this animal, the tumor metastasized to different organs and was suspected to be the cause of a pathological femoral fracture, while in our case the lungs were the only affected organ.

Bronchioalveolar adenocarcinomas are the most commonly diagnosed lung cancer in humans. The lepidic-predominant type is classified as grade 1 tumors as it is less invasive than the other (acinar, solid, papillary, and micropapillary) subtypes [8, 9]. Lepidic growth means, that neoplastic cells spread by creeping along the airway epithelium rather than growing exponentially outward. In humans, the prognosis after treatment by surgical resection, chemotherapy, radiation therapy or combinations is better than in other types. No specific risk factors for this type of lung neoplasia are known [8, 10]. In the described case, no history of a previous lung affection was reported.

To our knowledge, there is no description of radiological findings in cases of bronchioalveolar tumors in SAC. Gall, Zekas, Van Metre, & Holt reported radiological findings of two cases of metastatic tumors in SAC [11]. These cases showed a diffuse miliary pattern in one animal and amore atypical, unstructured interstitial pattern in the second. The findings were, according to the authors, consistent with the appearance of pulmonal metastasis in other species.

In dogs, the typical radiographic appearance of primary pulmonary neoplasia of all types is a solitary, well-defined nodule. Other patterns can also be seen and include multiple nodules, homogenous lobar consolidation, disseminated reticulonodular, or mixed alveolar interstitial patterns. Bronchioalveolar carcinomas may appear as a mixed pattern of irregularly shaped nodules with bronchial cuffs. Lobar consolidation may also be seen due to inflammation and tumor invasion causing atelectasis of affected lobes [12]. The described case seemed to show some of the mentioned pathological findings above, but a definitive diagnosis was not possible.

The CT-scan was helpful to get a better overview of the condition of the lungs, but the classification of the lesions remained difficult due to the lack of experience with these species. The only study describing non-pathological CT-findings in SAC is reported by Cooley et al. in 2013 [13]. No literature describing pathological findings was available. Regarding the lack of knowledge of the appearance of pulmonary disease of alpacas in CT-scans, a comparison with other domestic species was made. In CT images of dogs, primary pulmonary tumors appeared bronchocentric of origin, which was also the case in the alpaca, but most of the lesions were singular and well circumscribed [14], while in the described case multiple, ill-defined lesion were present. In cats, lung tumors appear in the CT-scan often as masses with irregular margins [15] and are therefore more comparable to the lesions seen in the alpaca.

As the radiological findings in the alpaca mare were not typical and only few publications on radiological pathology of camelids are available [11], a definitive diagnosis intra vitam was not possible in this case. Differentials as tuberculosis and fungal granulomas could not be excluded, as they can lead to similar radiological and clinical findings [16, 17]. As an area with pathological findings was identified by ultrasonographic examination, a punction, fine needle aspiration or biopsy and following cytological examination would have been possible and could have led to a diagnosis on the living animal. In cases of unclear radiological findings it is important to consider this diagnostic option [13].In the present case the owner elected euthanasia of his animal and therefore this examination was not performed.

This report describes the clinical, radiological and pathological findings of a lepidic-predominant bronchioalveolar adenocarcinoma in an adult alpaca mare. For the first time, radiological and CT findings of a lepidic-predominant bronchioalveolar adenocarcinoma in a SAC can be provided. A better knowledge of the appearance of pulmonary neoplasia in camelids can help to differentiate between pulmonary neoplasia and similar pulmonary lesions as granulomas on thoracic radiography and CT-imaging. This would also enable choices of appropriate further diagnostic tests and determine probable prognosis. Since SACs are increasingly gaining in importance as pets and their owners are therefore usually willing to invest more in their diagnostics and therapy, it is important to gain experience in appropriate diagnostic options.

## Abbreviations

CT: Computed tomography
SAC: South American camelid
